# Sequestosome 1 Is Part of the Interaction Network of VAPB

**DOI:** 10.3390/ijms222413271

**Published:** 2021-12-09

**Authors:** Christina James, Christof Lenz, Henning Urlaub, Ralph H. Kehlenbach

**Affiliations:** 1 Department of Molecular Biology, Faculty of Medicine, GZMB (Göttinger Zentrum für Molekulare Biowissenschaften), Georg-August-University Göttingen, Humboldtallee 23, 37073 Göttingen, Germany; 2 Bioanalytics Group, Institute of Clinical Chemistry, University Medical Center Göttingen, Robert-Koch-Straße 40, 37075 Göttingen, Germany; christof.lenz@med.uni-goettingen.de (C.L.); henning.urlaub@mpibpc.mpg.de (H.U.); 3 Bioanalytical Mass Spectrometry Group, Max Planck Institute for Biophysical Chemistry, Am Fassberg 11, 37077 Göttingen, Germany

**Keywords:** VAPB, amyotrophic lateral sclerosis, SQSTM1, sequestosome, p62

## Abstract

VAPB (Vesicle-Associated-membrane Protein-associated protein B) is a tail-anchored membrane protein of the endoplasmic reticulum that can also be detected at the inner nuclear membrane. As a component of many contact sites between the endoplasmic reticulum and other organelles, VAPB is engaged in multiple protein interactions with a plethora of binding partners. A mutant version of VAPB, P56S-VAPB, which results from a single point mutation, is involved in a familial form of amyotrophic lateral sclerosis (ALS8). We performed RAPIDS (rapamycin- and APEX-dependent identification of proteins by SILAC) to identify proteins that interact with or are in close proximity to P56S-VAPB. The mutation abrogates the interaction of VAPB with many known binding partners. Here, we identify Sequestosome 1 (SQSTM1), a well-known autophagic adapter protein, as a major interaction/proximity partner of P56S-VAPB. Remarkably, not only the mutant protein, but also wild-type VAPB interacts with SQSTM1, as shown by proximity ligation assays and co-immunoprecipiation experiments.

## 1. Introduction

**Figure 1:**
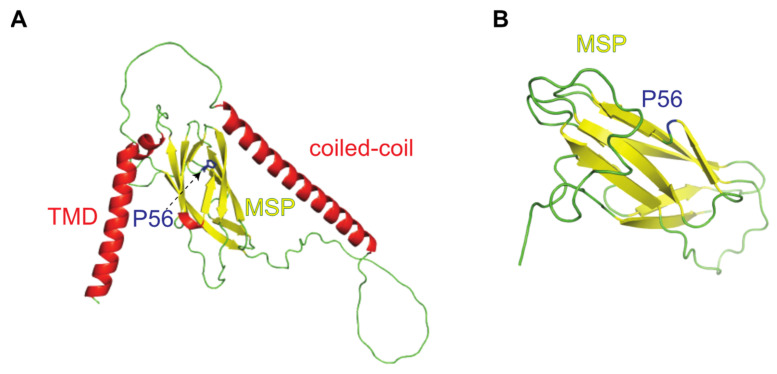
Structure of VAPB. (**A**) Structure prediction by AlphaFold [14]. The coiled-coil domain and the transmembrane domain (TMD) are represented as ribbons (red). The MSP domain with seven β-sheets is shown in yellow. (**B**) Crystal structure of the human MSP domain of VAPB (PDB entry 3IKK [15]). Amino acid residue P56 is depicted in blue.

The Vesicle-Associated-membrane Protein-associated protein B (VAPB) is a member of a small protein family that also contains VAPA and the MOtile SPerm Domain- containing proteins 1, 2 and 3 (MOSPD1, MOSPD2 and MOSPD3) [1,2]. These proteins share a Major Sperm Protein (MSP) domain that is involved in protein–protein interactions. The crystal structure of the MSP domain of VAPA has been solved, revealing a seven-stranded immunoglobulin-like β sandwich (Figure 1; [3]). In many cases, the MSP-domain mediates binding to so-called FFAT (two phenylalanines in an acidic tract) motifs in the partner protein [4,5,6,7,8]), facilitating the formation of membrane contact sites between two organelles [9]. VAPA, VAPB and MOSPD2 contain one transmembrane domain (TMD) close to their C-terminal ends, whereas MOSPD1 and MOSPD3 contain a second, adjacent TMD. For VAPB, the insertion of the protein into the cellular membrane system has been shown to require post-translational mechanisms [10]. After insertion, the protein largely localizes to the endoplasmic reticulum (ER) but can also be detected at the level of the inner nuclear membrane (INM; [11,12]). VAPA and VAPB can form homo- or heterodimers, where predicted coiled-coil regions but also the transmembrane domains contribute to oligomerization [13]. Structures of the entire VAPB protein, according to an AlphaFold prediction [14] and of the MSP-domain of VAPB [15], are shown in Figure 1A,B, respectively.

In the past few years, several proteomic screens have established the interaction network of VAPB, with more than 200 potential binding partners [1,11,16,17]. Prominent interaction partners include the oxysterol binding proteins (OSBPs), which are components of contact sites between the ER and the Golgi complex that play a role in lipid transfer [18] and the acyl-CoA binding domain protein ACBD5 that promotes the interaction of the ER with peroxisomes [19]. The large repertoire of VAPB-interacting proteins explains the multitude of functions that have been assigned to VAPB, e.g., in lipid transfer [20], calcium homeostasis [6], membrane trafficking [21,22], microtubule organization [5] and the unfolded protein response (UPR; [23,24]).

ALS is a progressive neurodegenerative disease causing the loss of upper and lower motoneurons and unclear pathomechanisms [25,26,27]. A point mutation in the VAPB gene, resulting in an exchange of proline 56 within the MSP domain to serine (P56S-VAPB), has been linked to a familiar, autosomal-dominant form of amyotrophic lateral sclerosis (ALS8) [28]. The structural consequences of the P56S point mutation, which would affect a distinct kink between two β strands within the MSP-domain (Figure 1B), have already been identified in the original paper by Nishimura et al. [28] describing the mutation. Subsequently, it was shown that the mutation leads to the exposure of hydrophobic patches, facilitating the aggregation of the protein [13]. Structural analyses [15,29] then suggested that in the mutant the characteristic β-fold of the MSP domain is converted into a helical structure with amphiphilic segments that can be stabilized upon insertion into membranes. Through transfection experiments, it was confirmed that the P56S mutation in VAPB reduces the solubility of the protein and ultimately results in a localization of the protein in dot-like structures that appeared to be distinct from the ER [23]. The initial membrane insertion of P56S-VAPB, however, occurs at the level of the ER, similar to that of the wild type protein [10] and the aggregates observed in overexpressing cells may ultimately lead to extensive restructuring of the ER [10,30]. The P56S mutation also leads to a reduced UPR (unfolded protein response), a cellular pathway that would normally promote the degradation of misfolded and/or aggregated cellular proteins. On the other hand, P56S-VAPB is polyubiquitinated [31] and subject to proteasomal degradation [30]. With respect to the disease, it remains unclear whether it results from a gain- or a loss-of-function effect, based on the P56S-VAPB mutation [32]. Specifically, reduced expression levels of the wild-type protein from a single allele could lead to a loss-of-function effect. Furthermore, the mutant protein can sequester the wild-type protein in insoluble aggregates [13,23,31], leading to its removal from the active pool of proteins that could engage in functional interactions. It is clear that reduced protein–protein interactions at many levels could contribute to ALS8 as well. A gain-of-function effect, on the other hand, could result from aberrant interactions of P56S-VAPB that are normally not observed for the wild-type protein. Several proteins have been reported to recruit to P56S-VAPB-aggregates, including ACBD5 [33], the HCN2 channel [34], the Yip1-interacting factor homologue A (YIF1A; [35]), stress granule markers such as TIAR1 or G3BP [36] and the ubiquitin-binding protein sequestosome 1 [36].

Besides its major localization at the ER, VAPB can also be found at the INM [11,12]. Interestingly, the P56S mutation in VAPB was shown to lead to changes in the organization of the nuclear envelope and Tran, Ngsee and coworkers suggested defects in the transport of nucleoporins (e.g., Nup214) and of proteins of the INM [37]. Here, we reinvestigate the effects of P56S-VAPB on the nuclear envelope and its possible targeting of the INM to search for possible interaction partners of the mutant protein in an unbiased proteomic approach.

## 2. Results

### 2.1. Effects of P56S-VAPB on Proteins of the Nuclear Envelope

**Figure 2:**
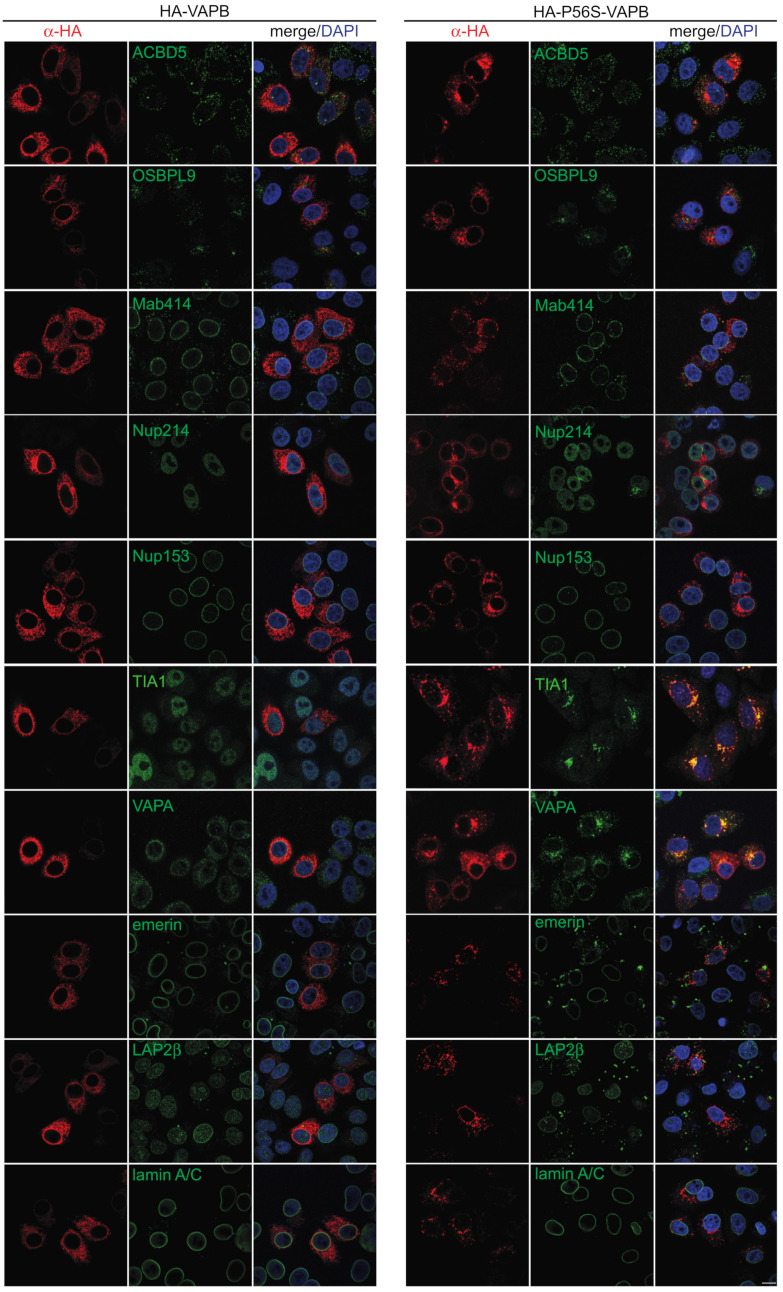
Mutant P56S-VAPB affects the subcellular localization of a subset of proteins. HeLa cells were transfected with plasmids coding for HA-tagged wild type (HA-VAPB) or mutant (HA-P56S-VAPB) VAPB and analyzed by indirect immunofluorescence and confocal microscopy detecting the HA-tagged proteins and endogenous ACDB5, OSBPL9, FG-Nups (using the Mab414 antibody), Nup214, Nup153, TIA1, VAPA, emerin, LAP2β and lamin A/C as indicated. Bar, 10 µm.

The mutant P56S-VAPB has previously been shown to sequester several RNA binding proteins including FUS and TDP-43 in transfected cells [36]. It also causes nuclear envelope defects with effects on proteins of the inner nuclear membrane and the NPC (nuclear pore complex) [37]. We re-investigated these effects and expressed HA-tagged wild-type and mutant VAPB in HeLa cells. The subcellular localization of several candidate proteins that had been suggested to interact with wild type or mutant VAPB was then analyzed by fluorescence microscopy (Figure 2). A very clear effect was observed for the RNA-binding protein TIA1, which accumulated inside the nucleus in control cells or in cells expressing wild type HA-VAPB, but co-localized with HA-P56S-VAPB in cytoplasmic aggregates of transfected cells. This recruitment to cytoplasmic aggregates was also observed for endogenous VAPA, as described previously [38]. However, we did not detect the recruitment of FUS or TDP-43 to the cytoplasmic aggregates, in contrast to a previous publication [36] and no colocalization with autophagosome or lysosome membrane proteins, LC3 and LAMP1 was observed in our study (Figure S1). Furthermore, two established interaction partners of VAPB, ACBD5 and OSBPL9 were not affected by the P56S mutation (Figure 2). Likewise, several nucleoporins (Nup153 and Nup214 (Figure 2) and ELYS and Nup358 (Figure S1)) did not change their subcellular localization in cells expressing HA-P56S-VAPB. The INM-proteins emerin and LAP2β, by contrast, were largely lost from the NE, showed no clear colocalizaton with HA-P56S-VAPB and were detected in aggregate-like structures (Figure 2). Staining with antibodies against several FG-nucleoporins (Mab414) or lamins A/C revealed that the overall structure of the NE was not compromised in cells expressing HA-P56S-VAPB. Together, these results suggest that the amino acid exchange at position 56 (P56S) can have different effects with respect to protein–protein interactions, such that (i) some interactions (e.g., with VAPA) are retained. (ii) Others are perhaps lost (e.g., with OSBPL9 or ACBD5) or (iii) could be newly established (e.g., with TIA1).

### 2.2. Identification of Interaction Partners of P56S-VAPB

**Figure 3:**
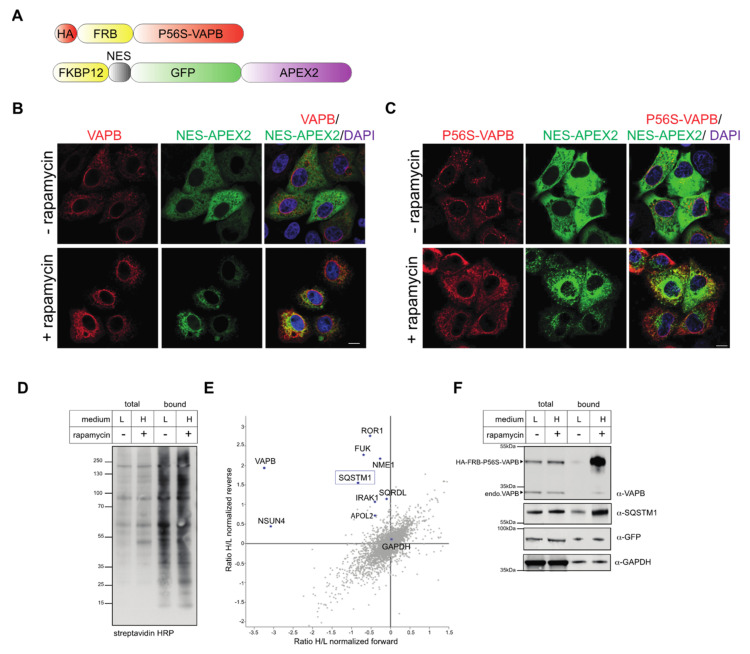
RAPIDS with P56S-VAPB and an NES-APEX2 reporter. (**A**) Scheme of HA-FRB-P56S-VAPB and FKBP12-NES-GFP-APEX2. (**B**,**C**) HeLa cells were cotransfected with plasmids coding for HA-FRB-VAPB (VAPB (**B**) or HA-FRB-P56S-VAPB (P56S-VAPB (**C**)) and FKBP12-NES-GFP-APEX2 (NES-APEX2) and treated with or without rapamycin, as indicated. The localization of the overexpressed proteins was detected by indirect immunofluorescence and confocal microscopy using an antibody against the HA-tag (red) or directly, via the GFP-signal (green). In the merged pictures (right) the DAPI signal is also shown. Bar, 10 μm. (**D**–**F**) HA-FRB-P56S-VAPB-transfected cells were grown in “light” (L) or “heavy” (H) medium as indicated and subjected to APEX2-dependent biotinylation in the absence (−) or presence (+) of rapamycin in forward and reverse experiments. (**D**) Cells were lyzed, proteins were bound to Neutravidin beads and the total and the bound fractions were analyzed by Western-blotting, using streptavidin-HRP for detection. (**E**) Scatter-plot showing normalized log2-ratios of proteins eluted from Neutravidin beads in forward and reverse experiments. Specifically biotinylated proteins are expected in the upper left quadrant. (**F**) Total and bound proteins were analyzed as in (**C**) using specific antibodies against VAPB, SQSTM1, GFP and GAPDH.

We have previously used RAPIDS (rapamycin- and APEX-dependent identification of proteins by SILAC) to investigate the “proximome” of wild type VAPB [11] and the inner nuclear membrane protein emerin [39]. This method combines the rapamycin-dependent dimerization of proteins [40] with the biotinylation of proteins in close proximity to ascorbate peroxidase 2 (APEX2), an enzyme that generates phenoxyl radicals from biotin phenol [41,42]. Compared to the classic APEX-approach, RAPIDS offers the advantage of an easy identification of specifically biotinylated proteins. Briefly, cells are co-transfected with constructs coding for a protein of interest and for a GFP-tagged version of APEX2, both containing a rapamycin-dependent dimerization cassette (an FRB- and an FKBP12-domain, respectively). SILAC (stable isotope labeling with amino acids in cell culture) then allows for a quantitative comparison of biotinylated proteins (i.e., proteins in close proximity to the protein of interest) in the absence or presence of rapamycin. We then applied the same approach to identify possible interaction and/or the proximity partners of the mutant P56S-VAPB. Cells were co-transfected with HA-tagged FRB-P56S-VAPB (and for comparison also with wild-type HA-FRB-VAPB) and different versions of APEX2. In a first set of experiments, we used a version of the enzyme that contains a nuclear export sequence (FKBP12-NES-GFP-APEX2; Figure 3A) and is therefore largely excluded from the nucleus. As shown in Figure 3B, FKBP12-NES-GFP-APEX2 partially colocalized with HA-FRB-VAPB upon addition of rapamycin. As seen before, P56S-VAPB (here: HA-FRB-P56S-VAPB) localized to the cytoplasm with some characteristic aggregates (Figure 3C). Upon the addition of rapamycin to the cells, the green reporter protein was partially recruited to these aggregates, demonstrating a drug-induced dimerization of the two proteins. Transfected cells that were incubated with or without rapamycin were treated with biotin-phenol and H_2_O_2_ to induce an APEX2-dependent modification of proteins. Biotinylated proteins were then captured using neutravidin beads and analyzed by SDS-PAGE. The overall level of proteins captured on the neutravidin beads did not change significantly upon the addition of the drug (Figure 3D), suggesting that only a subset of individual proteins was modified. Based on these results, we decided to subject the bound fraction, (i.e., proteins recovered from the neutravidin beads) to quantitative mass spectrometry. For RAPIDS, the cells had initially been grown in SILAC-media (with light or heavy isotopes of lysine and arginine) and then treated with or without rapamycin, respectively. To increase the specificity, a label-swap experiment was also performed. In Figure 3E, proteins that are specifically biotinylated upon the addition of rapamycin are expected to appear in the upper left quadrant of the graph. Proteins that are not affected (such as GAPDH) should be positioned within the cloud, centered around the intersection of the *X*- and *Y*-axes. As expected, VAPB itself was the most significant protein found to be biotinylated in this analysis, in line with the result of the Western blot analysis. As shown in Figure 3E, treatment with rapamycin also resulted in high levels of biotinylated HA-FRB-P56S-VAPB. Additionally, FKBP12-NES-GFP-APEX2 was recovered in the bound fractions derived from cells treated with or without rapamycin, probably due to the rapamycin-independent biotinylation of the enzyme itself. Likewise, the level of bound GAPDH did not change upon the addition of rapamycin. One of the few proteins also detected in the upper left quadrant was sequestosome 1, also known as SQSTM1 or p62 [43,44]. As a specificity control, we also subjected cells to RAPIDS (plus/minus rapamycin) that had only been transfected with FKBP12-NES-GFP-APEX2. Under these conditions, endogenous proteins could become specifically biotinylated through the addition of rapamycin, independently of an exogenous, FRB-containing fusion protein. The results of this experiment are shown in Figure S2. Importantly, the few identified proteins (upper left quadrant in Figure S2D) were distinct from those found in Figure 3.

**Figure 4:**
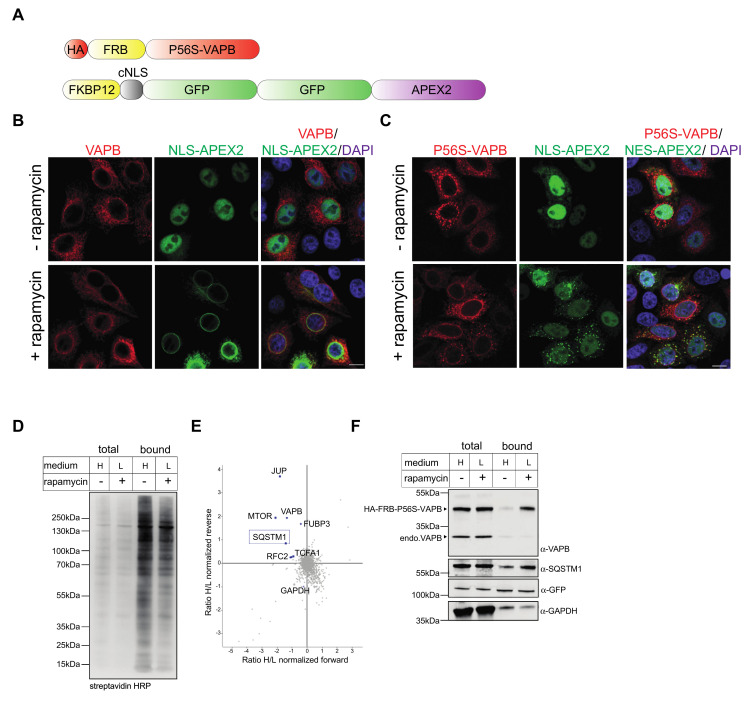
RAPIDS with P56S-VAPB and an NLS-APEX2 reporter. (**A**) Scheme of HA-FRB-P56S-VAPB and FKBP12-cNLS-GFP_2_-APEX2. (**B**,**C**) HeLa cells were cotransfected with plasmids coding for HA-FRB-VAPB (VAPB (**B**)) or HA-FRB-P56S-VAPB (P56S-VAPB (**C**)) and FKBP12-cNLS-GFP_2_-APEX2 (NLS-APEX2) treated with or without rapamycin, as indicated. The localization of the overexpressed proteins was detected by indirect immunofluorescence followed by confocal microscopy using antibodies against the HA-tag (red) or directly, via the GFP-signal (green). In the merged pictures (right), the DAPI signal is also shown. Bar, 10 μm. (**D**–**F**) HA-FRB-P56S-VAPB-transfected cells were grown in “light” (L) or “heavy” (H) medium as indicated and subjected to APEX2-dependent biotinylation in the absence (−) or presence (+) of rapamycin in forward and reverse experiments. (**D**) Cells were lyzed, proteins from cell lysates were bound to Neutravidin beads and the total and the bound fractions were analyzed by Western-blotting, using streptavidin-HRP for detection. (**E**) Scatter-plot showing normalized log2-ratios of proteins eluted from Neutravidin beads in forward and reverse experiments. (**F**) Total and bound proteins were analyzed as in (**D**), using specific antibodies against VAPB, SQSTM1, GFP and GAPDH.

Compared to our previous study, in which we analyzed wild type VAPB [11], only a few proteins were found as potential proximity partners of P56S-VAPB. We therefore modified our approach and used a version of the enzyme (FKBP12-cNLS-GFP_2_-APEX2; Figure 4A) containing a classic nuclear localization signal that leads to an accumulation of the fusion protein in the nucleus. As in our previous study, this reporter protein was recruited to the nuclear envelope through the addition of rapamycin in cells co-expressing wild type HA-FRB-VAPB (Figure 4B) [11], suggesting a partial localization of the protein at the INM. In cells co-expressing HA-FRB-P56S-VAPB, a GFP-signal at the nuclear envelope was not observed upon addition of rapamycin (Figure 4C), suggesting that the mutant protein does not reach the INM, as previously shown for the wild type protein [11]. Instead, upon addition of rapamycin to the cells, FKBP12-cNLS-GFP_2_-APEX2 was partially recruited to the cytoplasmic aggregates that are typically observed for mutant versions of VAPB. Evidently, the GFP-reporter protein containing the nuclear localization signal is able to diffuse back into the cytoplasm where it can interact with its target. Hence, this should be a valid approach for the identification of proximity partners of HA-FRB-P56S-VAPB in the cytoplasm, a compartment in which the concentration of the APEX2-fusion protein is very low in the absence of rapamycin. Furthermore, the addition of rapamycin did not result in a marked increase of overall levels of biotinylated proteins (Figure 4D), as seen before (Figure 3D). Again, RAPIDS (again performed as a label-swap experiment) yielded a rather small set of proteins in the upper left quadrant (Figure 4E), suggesting that only a few proteins were specifically biotinylated through the addition of rapamycin. The only proteins that were identified in both approaches (Figure 3 and Figure 4) were VAPB itself and SQSTM1. Similar to the results shown in Figure 3F, the addition of rapamycin to the cells led to a clear increase of the level of HA-FRB-P56S-VAPB, as captured on neutravidin beads upon the addition of rapamycin, whereas the levels of GAPDH and FKBP12-cNLS-GFP_2_-APEX2 did not change significantly (Figure 4F). To corroborate the results of the mass spectrometry, we probed the Western blots (Figure 3F and Figure 4F) with specific antibodies against SQSTM1. Indeed, the addition of rapamycin resulted in a clear increase of SQSTM1 levels as captured on the neutravidin beads, confirming their specific biotinylation. Together, these results suggest that SQSTM1 is a prominent proximity/interaction partner of P56S-VAPB. However, SQSTM1 was not identified as an interaction/proximity partner of VAPB in our original screen [11]. Nevertheless, we also addressed the possibility of an interaction with the wild-type protein.

**Figure 5:**
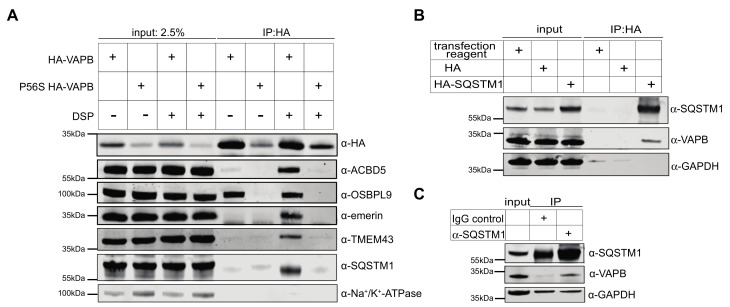
Coprecipitation of endogenous and overexpressed VAPB and SQSTM1. (**A**) HeLa cells were transfected with constructs coding for HA-VAPB or HA-P56S-VAPB and treated with (+) or without (−; DMSO as a control) the cross-linker DSP. Proteins from lysates were immunoprecipitated (IP) using anti-HA antibodies. After Western blotting, precipitated proteins were detected using antibodies against the HA-tag, ACBD5, OSBPL9, emerin, TMEM43, SQSTM1 and Na^+^/K^+^-ATPase as a loading control. (**B**) HeLa cells were transfected with an empty HA-vector (HA), a plasmid coding for HA-SQSTM1 or were treated with the transfection reagent alone. Proteins from cell lysates were immunoprecipitated using anti-HA antibodies. (**C**) Endogenous proteins from cell lysates were precipitated using rabbit anti-SQSTM1 and rabbit IgG as a control. Note that SQSTM1 and the IgG heavy chain, which is detected in the control reaction too, overlap. (**B**,**C**) Precipitated proteins were detected on Western blots using antibodies against SQSTM1, VAPB and GAPDH as a control.

First, we analyzed the possible interaction between VAPB/P56S-VAPB and SQSTM1 through co-immunoprecipitation experiments. Cells were transfected with constructs coding for HA-tagged versions of the wild type or the mutant proteins, and cell lysates were subjected to immunoprecipitation using anti-HA antibodies. Note that the observed protein levels of HA-P56S-VAPB are much lower than those of the wild-type version (Figure 5A). This could result from lower expression levels, reduced solubility and/or enhanced degradation of the mutant protein. To detect weak interaction partners, the experiments were performed in the absence or presence of the bifunctional crosslinker DSP (dithiobis(succinimidyl propionate)). As shown before [11], OSBPL9, a known interaction partner of VAPB [18], was detected as a co-precipitating protein from lysates of cells expressing wild type HA-VAPB, even in the absence of the crosslinker (Figure 5A). Other interaction/proximity partners such as ACBD5, TMEM43 and emerin required the addition of DSP. Interestingly, SQSTM1, which had not been originally identified by RAPIDS as a proximity partner of wild type VAPB [11], was also detected in the coprecipitate. On the other hand, HA-P56S-VAPB was detected at lower levels compared to HA-VAPB, probably due to reduced expression levels and/or reduced solubility [23] of the protein in our experimental conditions. None of the proteins that could readily be identified as direct or indirect binding partners of wild type VAPB coprecipitated with HA-P56S-VAPB, even in the presence of the crosslinker.

We also performed the reverse experiment and transfected cells with a construct coding for HA-SQSTM1. Here, endogenous VAPB coprecipitated with HA-SQSTM1 in a specific manner, as no signal was detected when the cells had been transfected with an empty vector or treated with the transfection reagent alone (Figure 5B). Next, we addressed the possibility of an interaction between endogenous VAPB and SQSTM1, i.e., without overexpressing one of the two proteins. As shown in Figure 5C, VAPB co-precipitated when antibodies against SQSTM1 were added to the reaction, but not in a control reaction with unspecific IgG. As in Figure 5A, no crosslinker was required to detect the VAPB-SQSTM1 interaction. Finally, we addressed the mode of interaction of VAPB with SQSTM1 and transfected cells with constructs for wild-type HA-VAPB or a version of the protein with a mutation in its MSP-domain (HA-K87D/M89D-VAPB), known to affect binding to FFAT-motifs [3]. As shown in Figure S3A, the mutation greatly reduced the binding of VAPB to endogenous OSBPL9, as expected. The binding of SQSTM1, by contrast, was hardly affected, suggesting that the interaction does not require a classic FFAT-motif. Together, these results show that SQSTM1 can indeed form a complex with wild-type VAPB. Whether this results from direct or indirect interactions remains to be investigated.

**Figure 6:**
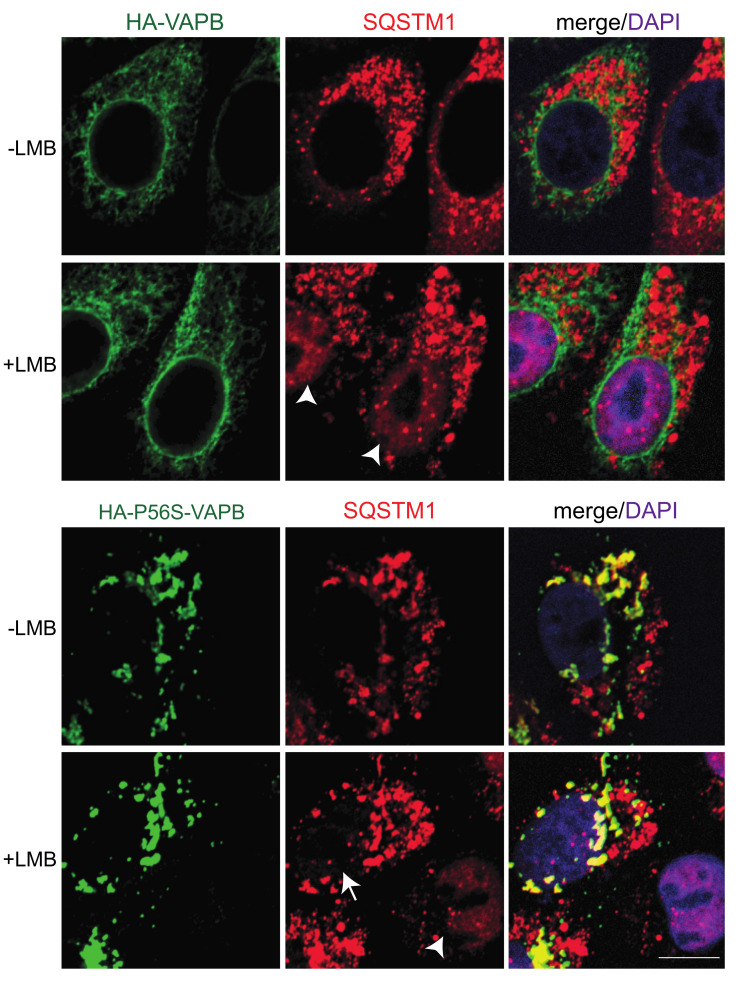
P56S-VAPB recruits endogenous SQSTM1 to cytoplasmic aggregates. HeLa cells were transfected with plasmids coding for HA-tagged wild type (HA-VAPB) or mutant (HA-P56S-VAPB) VAPB, and treated with (+) or without (−) LMB as indicated. Cells were subjected to indirect immunofluorescence using antibodies against the HA-tag and endogenous SQSTM1 and analyzed by confocal microscopy. For the merge, chromatin was stained with DAPI. Bar, 10 µm. Note that cells expressing HA-P56S-VAPB contain less SQSTM1 in the nucleus in the presence of LMB (arrow) compared to non-transfected cells or cells expressing wild type HA-VAPB (arrowheads).

Based on the biochemical results described above, we aimed to analyze the subcellular localization of endogenous SQSTM1 in cells expressing either HA-tagged wild type or mutant VAPB. HA-VAPB showed the characteristic ER-membrane pattern (Figure 6), as described above (Figure 2). However, SQSTM1 was excluded from the nucleus and exhibited a non-homogenous staining pattern that did not overlap with that of HA-VAPB. In cells expressing HA-P56S-VAPB, by contrast, a partial overlap of endogenous SQSTM1 with cytoplasmic aggregates of the mutant protein was obvious (Figure 6). We [45], and others [46] have previously identified SQSTM1 as a cargo protein of the nuclear export receptor CRM1. We therefore treated cells with the selective CRM1 inhibitor leptomycin B (LMB). As expected, this treatment resulted in the nuclear accumulation of SQSTM1 in control cells (Figure S3B) and in cells expressing wild type HA-VAPB (Figure 6). In cells expressing HA-P56S-VAPB, by contrast, SQSTM1 was not sequestered in the nucleus in the presence of LMB. Instead, it was retained at the cytoplasmic VAPB-aggregates, suggesting it to be an integral component of these structures, unable to move freely into the nucleus.

**Figure 7:**
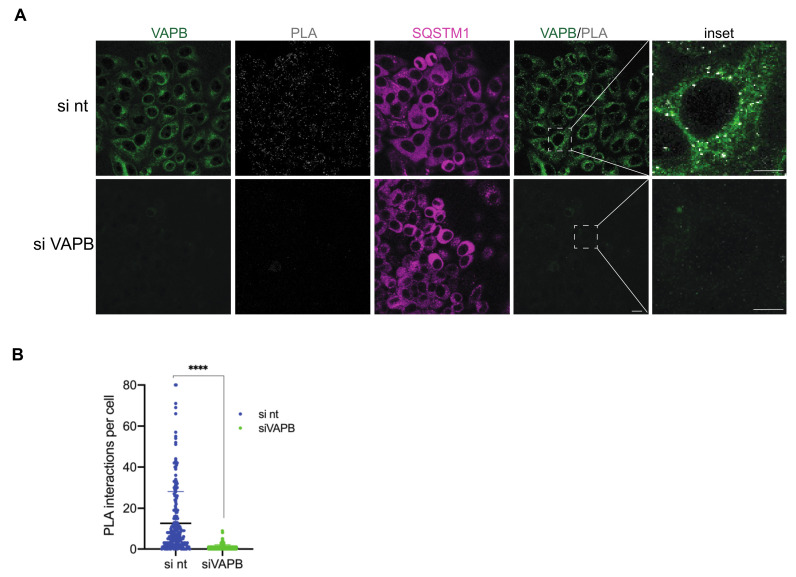
Endogenous VAPB is in close proximity to SQSTM1. HeLa cells were transfected with non-targeting (nt) siRNAs or siRNAs against VAPB and subjected to PLAs using antibodies against VAPB and SQSTM1. (**A**) VAPB and SQSTM1 were detected by indirect immunofluorescence and confocal microscopy. Bar, 10 µm. (**B**) Quantification of PLA interactions per cell analyzing a total of 250 cells per condition. The error bars correspond to the mean± S.D. from three independent experiments. ****, *p* < 0.0001.

As an independent approach, we used proximity ligation assays (PLA) [47] to address the interaction of endogenous VAPB and SQSTM1. In these assays, cells were fixed, and two proteins were decorated with specific antibodies. A possible interaction/proximity was then monitored using oligonucleotide-linked secondary antibodies that allow the generation of a fluorescent product in an amplification reaction. In our assay, with antibodies against endogenous VAPB and SQSTM1, we used cells that had been treated with siRNAs to deplete VAPB as a control. As shown in Figure 7, PLA-dots were readily detectable, mostly in the cytoplasm of cells. In VAPB-depleted cells, the number of PLA-dots was strongly reduced, suggesting a specific interaction/proximity of SQSTM1 and VAPB in cells that had been treated with non-targeting siRNAs. Collectively, our results show that SQSTM1 not only co-aggregates with mutant P56S-VAPB, but can also interact (directly or indirectly) with the wild type protein in situ.

**Figure 8:**
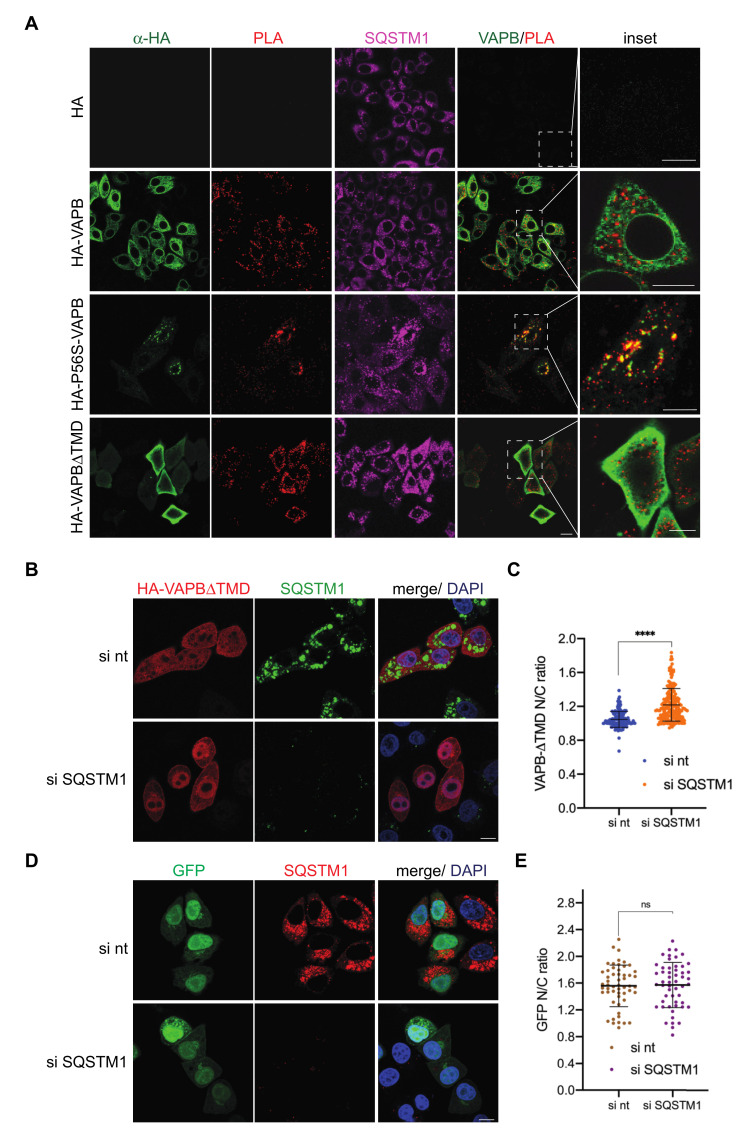
Exogenous VAPB (wild type, mutant and truncated versions) interact with endogenous SQSTM1. (**A**) HeLa cells were transfected with plasmids coding for an empty HA-vector (HA), HA-VAPB, HA-P56S-VAPB and HA-VAPB-∆TMD and subjected to PLAs using antibodies against the HA-tag and SQSTM1. Both SQSTM1 and versions of VAPB were detected by indirect immunofluorescence and confocal microscopy. Bar 10 µm. (**B**,**D**) HeLa cells were transfected with non-targeting (nt) siRNAs or siRNAs against SQSTM1 (si SQSTM1). After 24 h, the cells were transfected with a plasmid coding for HA-VAPB-∆TMD or GFP. SQSTM1 and HA-VAPB-∆TMD were detected using indirect immunofluorescence and confocal microscopy. Bar, 10 µm. (**C**,**E**) Quantification of nuclear to cytoplasmic (N/C) ratios of HA-VAPB∆TMD (**C**) and GFP (**E**) in cells treated with non-targeting (nt) siRNAs or siRNAs against SQSTM1 (si SQSTM1), analyzing a total of 200 cells (**C**) and 50 cells (**E**) per condition. The error bars correspond to the mean ± S.D. from three (**C**) and two (**E**) independent experiments. ****, *p* < 0.0001, ns, non-significant.

Finally, we performed PLAs with transfected cells to monitor possible interactions of endogenous SQSTM1 with HA-tagged versions of VAPB. As shown in Figure 8A, the combination of anti-SQSTM1- and anti-HA-antibodies yielded no PLA-signals in cells that had been transfected with an empty HA-vector. Specific signals could be detected, however, when the cells expressed wild-type HA-VAPB or P56S-VAPB. Interestingly, a VAPB version that lacked the transmembrane domain (VAPB-∆TMD) also resulted in specific PLA-dots in the cytoplasmic region of the transfected cells, suggesting that the membrane association of VAPB is not required for interaction with SQSTM1. We therefore compared the subcellular localization of HA-VAPB-∆TMD in control cells and in cells that had been treated with specific siRNAs to deplete SQSTM1. Strikingly, HA-VAPB-∆TMD, a protein of 25 kDa and therefore small enough to passively diffuse into and out of the nucleus, accumulated in the nucleus in the absence of SQSTM1 (Figure 8B,C). The subcellular localization of GFP, a protein of similar size as HA-VAPB-∆TMD, was not affected by the depletion of SQSTM1 (Figure 8D,E). Taken together, these results suggest that SQSTM1 is able to sequester the soluble fragment of VAPB in the cytoplasm. This result corroborates our findings of a direct or indirect interaction of wild type VAPB with SQSTM1.

## 3. Discussion

ALS is a devastating disease without an available cure. The majority of ALS cases are sporadic and cannot be linked to a distinct mutation. Nevertheless, a number of such mutations have been reported, e.g., in the genes coding for the RNA-binding proteins FUS (fused in sarcoma) and TDP-43 (TAR DNA- binding protein 43) [48]. For VAPB, which is an ER-membrane protein, the P56S-mutaion was the first to be described as a cause for ALS8, representing a familial form of the disease [28]. A few additional mutations in VAPB, besides the most common P56S mutation, have been reported as being linked to ALS [49,50,51]. Hence, a detailed understanding of the pathomechanisms that ultimately lead to ALS8 may help to better understand ALS in general.

Protein aggregation is a hallmark of several forms of ALS [26,48,52]. The P56S mutation in VAPB is known to greatly reduce the solubility of the protein [23] and to result in aggregation within membranes that are probably derived from the ER [30]. Through structural analyses suggesting that the MSP-domain cannot be folded in the mutant protein [15,29], many VAPB-interactions based on a classic FFAT motif in the interacting protein are expected to be perturbed. Indeed, the major proximity partners that we had originally observed for wild type VAPB [11] were not detected by RAPIDS with P56S-VAPB as a bait (Figure 3 and Figure 4). Accordingly, these proteins (e.g., ACBD5, OSBPL9, TMEM43 and emerin) could be cross-linked to the wild-type, but not the mutant protein in co-immunoprecipitation experiments (Figure 5A). VAPA, on the other hand, a protein that interacts with VAPB via coiled-coil regions and transmembrane domains [13], was recruited to cytoplasmic aggregates in cells expressing P56S-VAPB (Figure 2 and [53]), suggesting that this type of interaction is not perturbed by the mutation. VAPA, however, was not detected as a significant protein by RAPIDS, perhaps because of a lack of appropriate acceptor sites for biotinylation. The INM localization of both emerin and LAP2β was lost in P56S-VAPB transfected cells, suggesting a disruption in their transport to the INM, which was previously observed for emerin [37].

The major protein that was identified in both our RAPIDS approaches as a proximity partner of P56S-VAPB was sequestosome 1, also known as SQSTM1 or p62. Originally, SQSTM1 was identified as a protein that binds to SH2 domains of the protein kinase Lck and also interacts with ubiquitin [43,44]. Interestingly, mutant versions of SQSTM1 have also been implicated in certain forms of ALS [54,55]. Additionally, SQSTM1 is a multifunctional protein with many identified binding partners and acts as a cargo receptor in selective autophagy and other cellular pathways (for review see [56]). Since SQSTM1 is also involved in the mTOR (mechanistic target of rapamycin) pathway [57], it was important to show that it was not biotinylated in our control-RAPIDS, where the cells had been transfected with a construct only coding for FKBP12-NES-GFP-APEX2 (Figure S1). Evidently, biotinylation of SQSTM1 depended on the presence of P56S-VAPB in the transfected cells. A coaccumulation of SQSTM1 with P56S-VAPB aggregates has been shown very recently in atrophic muscle fibres as well as in skin fibroblasts from an ALS8-patient [36]. Furthermore, SQSTM1 colocalized with cytoplasmic P56S-VAPB inclusions in motor neurons derived from transgenic mice [58]. In an earlier study [30], a colocalization of the two proteins was not detected in a HeLa cell system [30], although a close juxtaposition could be observed.

This mutant version of VAPB is known to be subjected to polyubiquitination [23,30]. Hence, the identification of the ubiquitin-binding protein SQSTM1 [44] as a proximity partner of P56S-VAPB was not completely unexpected. Surprisingly, however, wild-type VAPB also interacts with SQSTM1, as shown for endogenous proteins by co-immunoprecipitation experiments (Figure 5) and PLAs (Figure 7). Furthermore, the soluble version of wild-type VAPB, VAPB-∆TMD, accumulated in the nucleus upon depletion of SQSTM1 by specific siRNAs, suggesting that it was partially sequestered in the cytoplasm by SQSTM1 in control cells (Figure 8B,C). This could result from the increased molecular weight of the VAPB-SQSTM1 complex that may not be able to diffuse passively through the NPC or by consolidating into immobile networks in the cytoplasm. Hence, we expect that SQSTM1 would also bind to the physiological form of VAPB that localizes to the ER or the INM. What then, is the biochemical basis for the observed interaction between VAPB and SQSTM1? The identification of SQSTM1 in a RAPIDS-approach with a version of VAPB containing a mutation that is known to reduce binding to classic FFAT-motifs [15,29], already suggested that other regions besides the MSP-domain could mediate the binding of VAPB to SQSTM1. This interpretation is corroborated by our finding that mutations in the MSP-domain of VAPB that abolish interactions with FFAT-motifs did not affect binding to SQSTM1 in co-immunoprecipitation experiments (Figure S3). Perhaps other regions in VAPB (e.g., the coiled-coil region (Figure 1A) are involved in VAPB-SQSTM1-interactions. To the best of our knowledge, SQSTM1 has not been previously demonstrated to associate with wild-type VAPB. We did not previously detect SQSTM1 as a potential interaction partner of wild-type VAPB in RAPIDS [11] and we can only speculate about possible reasons for this. One explanation is the preferential identification of selected membrane-associated proteins in such an approach, missing some of the established interaction partners [11]. Additionally, P56S-VAPB, which tends to aggregate, may sequester SQSTM1 to a sufficient level, thereby allowing biotinylation and its subsequent detection by mass-spectrometry. On the other hand, this aggregation is expected to reduce the solubility of the mutant protein, hampering the detection of SQSTM1 in co-immunoprecipitation experiments. We also addressed the interaction of proteins or protein fragments of VAPB and SQSTM1 expressed in bacteria, but failed to detect a strong binding (Figure S3C). Together, it appears likely that complex formation between VAPB and SQSTM1 requires additional components. One candidate for such a protein is the autophagosome scaffold protein FIP200, which interacts with SQSTM1 [59] as well as with VAPB [60]. Alternatively, the interaction might be rather transient and/or of a low affinity, allowing the detection of specific PLA-signals (Figure 7) but not of a clear colocalization by fluorescence microscopy (Figure 6). VAPB interactions might also require posttranslational modifications that are not present in proteins purified from bacteria. In cells expressing the mutant P56S-VAPB, on the other hand, their co-localization with SQSTM1 became evident and was readily detectable by confocal microscopy (Figure 6) and PLAs (Figure 8A).

At this point, we can only speculate about the physiological or pathophysiological consequences of the VAPB-SQSTM1 interaction. Similarly to FUS and TDP-43 [61], SQSTM1 could engage in heterotypic-phase separation [62,63], and thereby lead to the sequestration of additional proteins to the liquid phase. With respect to SQSTM1-function, P56S-VAPB could affect the role of the protein as an autophagy adapter protein [56]. Interestingly, VAPB has been previously implicated in the regulation of autophagy [64,65]. The molecular details of the VAPB-SQSTM1 interaction, however, remain to be investigated.

## 4. Materials and Methods

### 4.1. Plasmids

Standard procedures were used for cloning. To obtain APEX2-GFP_2_-NLS-FKBP12, the APEX2 coding sequence was amplified by PCR using pcDNA3-Connexin43-GFP-APEX2 (Addgene plasmid #49385) as a template and oligonucleotides 5′-TTTACTAGTATGGGAAAGTCTTACCCAACTGT and 5′-TTTACTAGTAAGGCATCAGCAAACCCAAG. The PCR product was cloned into a pEGFP-C1 derivative, encoding GFP_2_-cNLS-FKBP12 [66] via BcuI. For pcDNA3-FKBP12-NES-GFP-APEX2, the NES of HIV-1 Rev was amplified using oligonucleotides 5′-GCGGATCCGGGTCTATGGTGAGCAAGGGCGAGGAG and 5′-GCGGCGCGCCCTCCACAATCCTCGTTACAATCAAG and inserted into pcDNA3-FKBP12-GFP-APEX2 [11] via BamHI and AscI. To generate pEF-HA-FRB-P56S-VAPB, site-directed mutagenesis was performed using oligonucleotides 5′-GTAGGTACTGTGTGAGGTCCAACAGCGGAATCATC and 5′-GATGATTCCGCTGTTGGACCTCACACAGTACCTAC using pEF-HA-FRB-VAPB [11] as the starting vector. For pcDNA 3.1-HA-VAPB, the VAPB coding sequence was amplified by PCR using oligonucleotides 5′-CTTGGTACCGCGAAGGTGGAGCAGGTCC and 5′-GGATGGATCCCTACAAGGCAATCTTCCCAAT, using pmCherry-FRB-VAPB [11] as a template. The PCR product was cloned into pcDNA 3.1-HA via KpnI and BamHI. For pcDNA 3.1-HA-P56S-VAPB, proline to serine mutation at position 56 was introduced by site directed mutagenesis on pcDNA 3.1-HA-VAPB using oligonucleotides 5′-GTAGGTACTGTGTGAGGTCCAACAGCGGAATCATC and 5′-GATGATTCCGCTGTTGGACCTCACACAGTACCTAC. To generate pcDNA 3.1-HA-VAPB-∆TMD, the VAPB-∆TMD sequence was amplified by PCR using pmCherry-FRB-VAPB as a template and oligonucleotides 5′-CTTGGTACCGCGAAGGTGGAGCAGGTCC and 5′-TTTTGGATCCCTAGGTGCTAAGGCCTTCTTCC and cloned via KpnI and BamHI.

To generate pcDNA 3.1-SQSTM1-HA, SQSTM1 was PCR-amplified from pEGFP C1-SQSTM1 (obtained from Terje Johansen, Norway) and cloned into pcDNA3.1-HA via Hind111 and EcoR1. For pcDNA 3.1-HA-K87D/M89D-VAPB, site directed mutagenesis was performed using oligonucleotides 5′-gatcccaatgagaaaagtaaacacgattttatggttcagtctatgtttgct and 5′-ctagggttactcttttcatttgtgctaaaataccaagtcagatacaaacga using pcDNA3.1 HA-VAPB as a template. All plasmids were confirmed through sequencing.

### 4.2. Cell Culture and Transfections

HeLa P4 cells [67] were obtained from the NIH AIDS Reagent Program. Cells were maintained in DMEM (Life technologies, Carlsbad, CA, USA) with 10% (*v*/*v*) FBS (Thermo Fisher Scientific, Waltham, MA, USA), 100 U mL^−1^ penicillin, 100 µg mL^−1^ streptomycin and 2 mM L-glutamine (Thermo Fisher Scientific) in 5% CO_2_ at 37 °C. They were tested regularly for mycoplasma contamination.

For SILAC, cells were cultured in a medium containing heavy or light isotopes of arginine and lysine as previously described [11]. The incorporation rate was confirmed to be ≥ 97%.

Cells were transfected using the calcium phosphate method [68]. Lipofectamine RNAiMAX (Thermo Fisher Scientific, Waltham, MA, USA) was used for the siRNA mediated knockdown of VAPB and SQSTM1. VAPB siRNAs (5′-GCUCUUGGCUCUGGUGGUUUU, 5′-AAAACCACCAGAGCCAAGAGC) were purchased from Sigma. SMART pool siGenome SQSTM1 siRNAs (GAAAUGGGUCCACCAGGAA, GAUCUGCGAUGGCUGCAAU, GCAUUGAAGUUGAUAUCGA, GAAGUGGACCCGUCUACAG) and ON-Target plus non-targeting siRNA were obtained from Dharmacon (Lafayette, CO, USA, D-001810-01-50).

LMB (Enzo Life Sciences, Lörrach, Germany) was added to HeLa cells to a final concentration of 10 nM from a 10 µM stock in ethanol for 1 h.

### 4.3. Rapamycin-Dependent Biotinylation

HeLa P4 cells, grown in 10 cm dishes in a SILAC medium were subjected to biotinylation as described [11]. Briefly, the cells were transfected with pEF HA-FRB-P56S-VAPB/pcDNA3-FKBP12-NES-GFP-APEX2 or pEF-HA-FRB-P56SVAPB/pAPEX2-GFP_2_-NLS-FKBP12 and grown to confluency. A total of 500 µM biotin-phenol (Iris Biotech, Marktredwitz, Germany) was added to the cells, which were then incubated for 30 min, with or without 200 nM rapamycin (Sigma Aldrich, St. Louis, MO, USA). Experiments were performed in forward and reverse conditions [11]. After biotinylaton reactions, cells were washed twice with the quenching buffer (5 mM Trolox, 10 mM NaN_3_, 10 mM sodium ascorbate in PBS) and once with PBS. Cells used for indirect immunofluorescence were fixed with paraformaldehyde.

For Western blotting and SILAC analyses [11], cells were lysed in a RIPA buffer (50 mM Tris, pH 7.4, 5 mM Trolox, 0.5% (*w*/*v*) sodium deoxycholate, 150 mM NaCl, 0.1% (*w*/*v*) sodium dodecyl sulfate (SDS), 1% (*v*/*v*) Triton X-100, 1 mM phenylmethane sulfonyl fluoride (PMSF), 10 mM NaN_3_, 10 mM sodium ascorbate, 1 µg ml^−1^ aprotinin, 1 µg ml^−1^ leupeptin and 1 µg mL^−1^ pepstatin; 1 mL/dish). Biotinylated proteins were captured with Neutravidin beads (Thermo Fisher Scientific, Waltham, MA, USA). For each experiment, six batches of 130 µL Neutravidin beads were incubated in a 1 mL cell lysate overnight at 4 °C. The beads were washed once with washing buffer 1 (50 mM HEPES (pH 7.4), 0.1% (*w*/*v*) sodium deoxycholate, 1% (*v*/*v*) Triton X-100, 500 mM NaCl, 1 mM ethylenediaminetetraacetic acid (EDTA), once with washing buffer 2 (50 mM Tris (pH 8.0), 250 mM LiCl, 0.5% (*v*/*v*) Nonidet P-40, 0.5% (*w*/*v*) sodium deoxycholate, 1 mM EDTA) and twice with washing buffer 3 (50 mM Tris (pH 7.4) and 50 mM NaCl). Bound proteins were eluted from the beads and further analyzed as described [11].

### 4.4. Mass Spectrometric Analyses

After separation by SDS-PAGE, the reduction of proteins with dithiothreitol (DTT), alkylation with 2-iodoacetamide and digestion with trypsin [11], mass spectrometric analyses was performed as described [11]. The peptide mixtures were extracted, dried, reconstituted in 2% acetonitrile/0.1% formic acid/(*v*/*v*) and analysed by nanoLC-MS/MS on a hybrid quadrupole/orbitrap mass spectrometer (Q Exactive, Thermo Fisher Scientific, Dreieich, Germany) as described previously [69]. Raw data were processed using MaxQuant Software version 1.6.3.4 (Max Planck Institute for Biochemistry, Martinsried, Germany). Proteins were identified against the human reference proteome (v2020.02). For the statistical evaluation of relative protein quantitation values, the Perseus Software version 1.6.10.50 (Max Planck Institute for Biochemistry, Martinsried, Germany) was used and a two-sided Significance B test [70] was performed using normalized log_2_ ratios. A Benjamini-Hochberg correction was applied with a threshold value of 0.05. See Tables S1–S3 for results.

### 4.5. Data Availability

The MS proteomics data were deposited to the ProteomeXchange Consortium via the PRIDE [71] partner repository with the dataset identifier PXD029113.

### 4.6. Cross-Linking and Coimmunoprecipiation

Cross-linking and coimmunoprecipitation was performed as described [11]. Briefly, HeLa cells (2 million cells/dish) that were grown in three 10 cm dishes were transfected with plasmids coding for HA-VAPB or HA-P56S-VAPB. After transfection, the cells were washed with cold 1X PBS containing 0.1 mM CaCl_2_ and 1 mM MgCl_2_ and incubated with 1 mM dithiobis(succinimidyl propionate); (DSP, Thermo Scientific) in DMSO for 2 h. DMSO alone was used as a control. The cross-linker was quenched through the addition of 20 mM Tris-HCl, pH 7.4. After washing with PBS, cells were lysed with 1 mL lysis buffer (0.5% sodium deoxycholate, 50 mM Tris-HCl, pH 7.4, 150 mM NaCl, 0.25% SDS, and 0.5% Triton X-100 with protease inhibitors for 30 min on ice. The lysate was passed through a 27-gaugex3/4-inch needle and centrifuged at 15,000× *g* for 20 min at 4 °C. The supernatant was then incubated with anti-HA agarose beads (Sigma A2095) for 3 h at 4 °C. Proteins were eluted with a sample buffer containing 50 mM DTT.

For the immunoprecipitation of SQSTM1, HeLa cells were washed after 24 h of transfection with cold PBS and lysed with 1 mL lysis buffer (1% Triton X-100, 20 mM Tris-HCl, 150 mM NaCl, 2 mM DTT with complete protease inhibitor cocktail) for 30 min on ice. The lysate was centrifuged for 16,000× *g* for 15 min at 4 °C. The supernatant was then incubated with anti-HA agarose beads (Sigma A2095) for 3 h at 4 °C and proteins were eluted with the sample buffer.

For the immunoprecipitation of endogenous proteins, 4 µg of rabbit anti-SQSTM1, or IgG as control was immobilized on 75 µL Protein A-Sepharose 4 Fast Flow beads (GE Healthcare) for 3 h and then incubated in cell lysates lysed with 1 mL lysis buffer (1% Triton X-100, 20 mM Tris-HCl, 150 mM NaCl, 2 mM DTT with protease inhibitors) for 3 h. Proteins were eluted with the sample buffer.

### 4.7. Western Blot Analyses

Western blotting was performed according to standard methods using HRP-coupled or IRDye (LI-COR Biosciences) secondary antibodies (all antibodies are listed in Table S4). Biotinylated proteins were separated by SDS-PAGE, transferred to nitrocellulose, and blocked with 3% BSA in TBS-T (24.8 mM Tris, pH 7.4, 137 mM NaCl, 2.7 mM KCl, 1% (*v*/*v*) Tween 20) overnight at 4 °C. Membranes were incubated with streptavidin-HRP (Jackson ImmunoResearch Laboratories, West Grove, PA, USA; diluted 1:5000–1:40,000) for 1 h at room temperature and washed three times with TBS-T. To detect proteins, Immobilon Western Chemiluminescent HRP Substrate (Millipore, Burlington, MA, USA) or the LI-COR Odyssey Imaging System was used.

### 4.8. Immunofluorescence and Fluorescence Microscopy

For immunofluorescence, cells were fixed in 4% (*v*/*v*) paraformaldehyde, permeabilized with 0.5% (*v*/*v*) Triton X-100 in PBS for 5 min at room temperature and blocked with 3% (*w*/*v*) BSA or 0.2% fish gelatin (for detection of Nup214) in PBS for 20 min at room temperature. Staining was performed for 1 h using the appropriate antibodies (Table S4), diluted in 3% BSA or 0.2% fish gelatin in PBS. Cells were embedded in MOWIOL-DAPI and analyzed using an LSM510 Confocal laser scanning microscope using 63×/1.4 and 100×/1.4 oil immersion lenses (Zeiss, Oberkochen, Germany).

### 4.9. Proximity Ligation Assay (PLA)

For each well, 40,000 HeLa cells were seeded in 24-well plates, grown for 48 h, fixed with 4% paraformaldehyde and permeabilized with 0.05% (*v*/*v*) Triton X-100. PLA assays using the Duolink kit (Sigma Aldrich, St. Louis, MO, USA, DUO 9200) were have been described [11]. After blocking, cells were incubated with mouse anti-SQSTM1 and rabbit anti-VAPB and thereafter with the corresponding PLA probes. After ligation and amplification using the kit reagents, the cells were stained for SQSTM1 and VAPB and mounted with DAPI. Images were acquired using an LSM510 confocal laser scanning microscope with a 63×/1.4 oil immersion lens. In total, 250 cells from three independent experiments were analysed using CellProfiler [72]. An unpaired *t*-test was used for statistical analysis with a confidence interval of 95%.

### 4.10. Image Analysis

Cell Profiler [73] was used for the analysis of microscopic images. Nuclear-to-cytoplasmic ratios were quantified using a pipeline to measure fluorescence intensities of the nucleus and the cytoplasm. Nuclei were identified using the DAPI channel, setting their diameter to 40–200 pixels. Transfected cells were identified using the 594 nm channel, setting the diameter to 150–600 pixels, following a thresholding strategy. The cell area was defined by the watershed-image method. The cytoplasm was defined by subtracting the nuclear area from the cell area. Intensities of 594 nm signals were measured in the nucleus and the cytoplasm and nuclear-to-cytoplasmic signal intensity (N/C) ratios were calculated.

PLA signals were quantified following a similar pipeline in which PLA dots were identified using the 594 nm channel, setting the diameter at 2–10 pixels. A statistical analysis was performed using GraphPad Prism 8.

## Data Availability

Data is contained within the article or supplementary material.

## Supplementary Materials

The following are available online at https://www.mdpi.com/article/10.3390/ijms222413271/s1.
