# Supplementary material for: Sequestosome 1 Is Part of the Interaction Network of VAPB
Source: Int J Mol Sci. 2021 Dec 9;22(24):13271. doi: 10.3390/ijms222413271 (PMC8707790; doi:10.3390/ijms222413271)
Supplement: Supplementary file 1 [file ijms-22-13271-s001.zip › ijms-1442113-supplementary/ijms-1442113-supplementary-for publish.pdf]

International Journal of *Molecular Sciences*

MDPI

Supplementary Materials

Sequestosome 1 Is Part of the Interaction Network of VAPB

Christina James [1,*], Christof Lenz [2], Henning Urlaub [2,3] and Ralph H. Kehlenbach [1,*]

1 Department of Molecular Biology, Faculty of Medicine, GZMB (Göttinger Zentrum für Molekulare Biowissenschaften), Georg-August-University Göttingen, Humboldtallee 23, 37073 Göttingen, Germany
2 Bioanalytics Group, Institute of Clinical Chemistry, University Medical Center Göttingen, Robert-Koch-Straße 40, 37075 Göttingen, Germany; christof.lenz@med.uni-goettingen.de (C.L.); henning.urlaub@mpibpc.mpg.de (H.U.)
3 Bioanalytical Mass Spectrometry Group, Max Planck Institute for Biophysical Chemistry, Am Fassberg 11, 37077 Göttingen, Germany
* Correspondence: christina.james@med.uni-goettingen.de (C.J.); rkehlen@gwdg.de (R.H.K.)

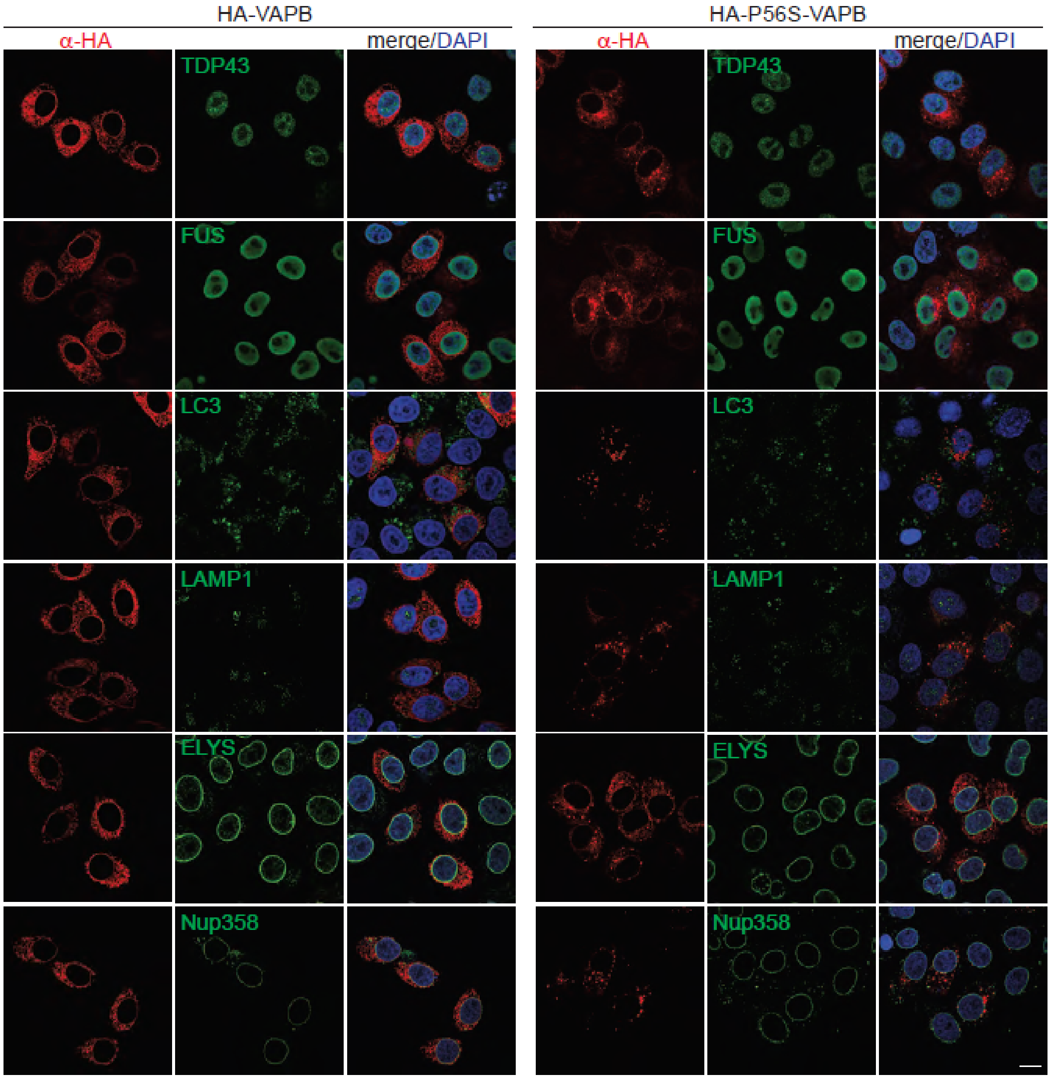

Figure S1. P56S-VAPB does not affect the localization of selected RNA bindind proteins, proteins involved in autophagy or nucleoporins. HeLa cells were transfected with either wild type (HA-VAPB) or mutant (HA-P56S-VAPB) VAPB and analyzed by indirect immunofluorescence and confocal microscopy detecting the HA-tagged proteins and endogenous TDP43, FUS, LC3, LAMP1, ELYS, Nup358, as indicated. Bar, 10 µm.

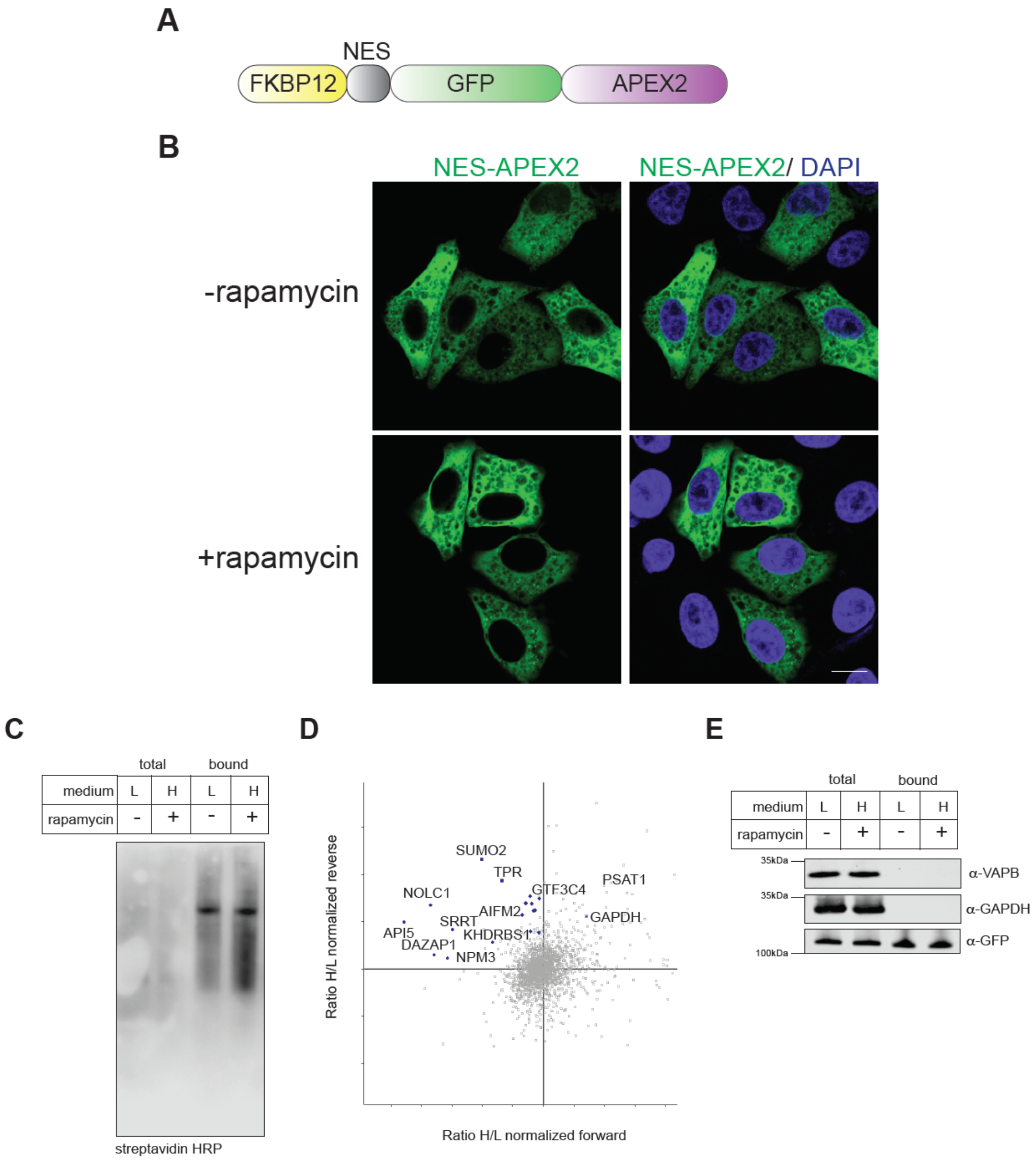

Figure S2. RAPIDS with NES-APEX2 reporter. (**A**) Scheme of FKBP12-NES-GFP-APEX2. (**B**) HeLa cells were transfected with a plasmid coding for FKBP12-NES-GFP-APEX2 (NES-APEX2) and treated with or without rapamycin, as indicated. The subcellular localization of the overexpressed proteins was analyzed by confocal microscopy, detecting the GFP-signal. In the merged pictures (right) also the DAPI signal is shown. Bar, 10 µm. (**C–E**) FKBP12-NES-GFP-APEX2 -transfected cells were grown in "light" (L) or "heavy" (H) medium as indicated and subjected to APEX2-dependent biotinylation in the absence (-) or presence (+) of rapamycin in forward and reverse experiments. (**C**) Proteins from cell lysates were bound to Neutravidin beads and the total and the bound fractions were analyzed by Western-blotting, using streptavidin-HRP for the detection of biotinylated proteins. (**D**) The scatter-plot shows normalized log2-ratios of proteins eluted from Neutravidin beads in forward and reverse experiments. (**E**) Total and bound proteins were analyzed using specific antibodies against VAPB, GFP and GAPDH.

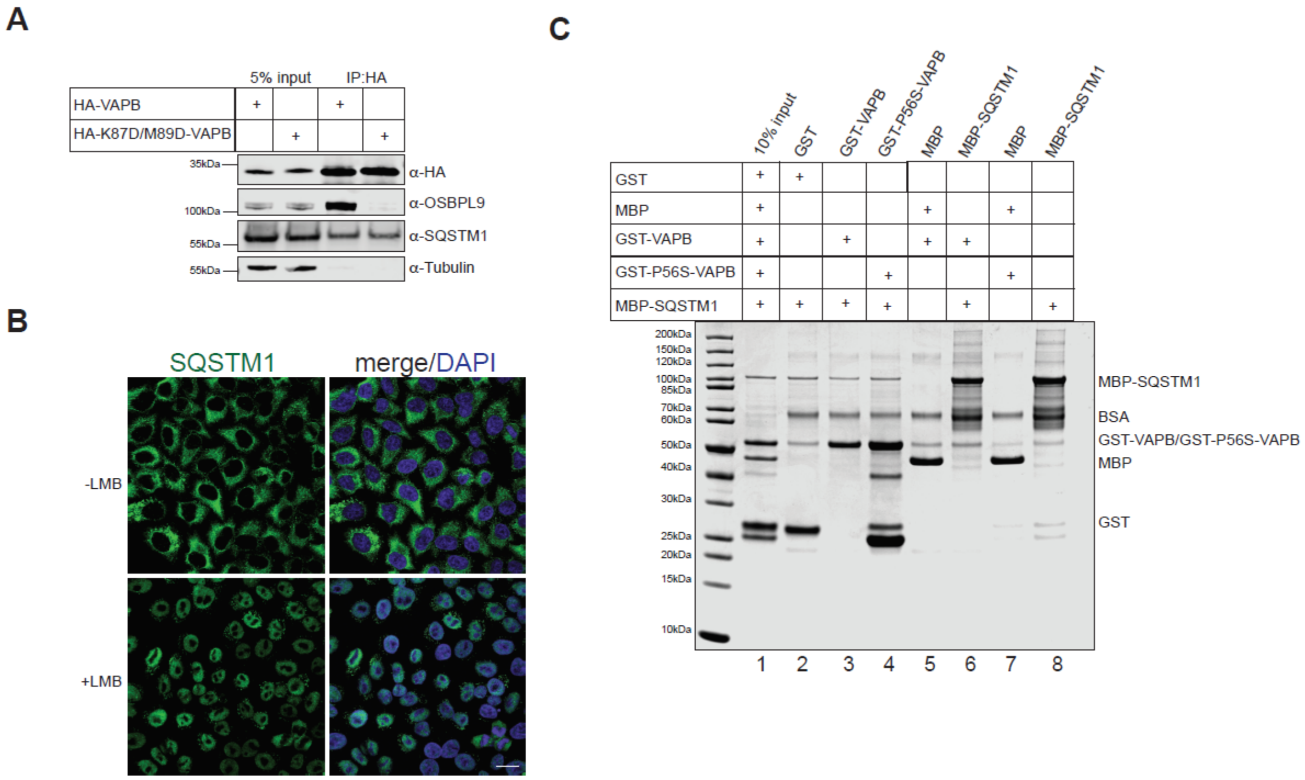

Figure S3. Analysis of VAPB-SQSTM1 interactions. (**A**) HeLa cells were transfected with either wild type (HA-VAPB) or the MSP-domain mutant (HA-K87D/M89D-VAPB) VAPB and subjected to immunoprecipitation assays using anti-HA antibodies. Co-precipitated proteins were detected using anti-SQSTM1 and anti-OSBPL9 antibodies. Tubulin was used as a loading control. (**B**) HeLa cells were treated with or without LMB for 1 h and endogenous SQSTM1 was detected using indirect immunofluorescence and confocal microscopy. Bar, 10 µm. (**C**) MBP-SQSTM1 or MBP alone were immobilized using amylose beads and incubated in the presence of either GST alone or GST-VAPB or GST-P56S-VAPB as indicated (lanes 2–4). GST proteins were immobilized using Glutathione Sepharose beads and incubated in the presence of either MBP alone or MBP-SQSTM1 as indicated (lanes 5–8). For generating pMal-SQSTM1 the sequence coding for SQSTM1 was cloned into the pMal-C2 vector. Transmembrane domain-lacking versions of VAPB or P56S-VAPB were cloned into pGEX-6p1 to obtain pGEX-VAPB or pGEX-P56S-VAPB. The recombinant proteins were purified using standard methods.
