# Supplementary material for: Sequestosome 1 Is Part of the Interaction Network of VAPB
Source: Int J Mol Sci. 2021 Dec 9;22(24):13271. doi: 10.3390/ijms222413271 (PMC8707790; doi:10.3390/ijms222413271)
Supplement: Supplementary file 1 [file ijms-22-13271-s001.zip › ijms-1442113-supplementary/TableS4_ list of antibodies used.pdf]

Table S4
Antibodies

Name	Species	Origin	Application	Dilution
**Primary antibodies**				
α-emerin	rabbit	#10351-1-AP, Proteintech	immunofluorescence	1:200
α-GFP	rat	#3H9, ChromoTek	Western blotting	1:1,000
α-laminA/C	mouse	# ab40567, Abcam	immunofluorescence	1:100
α-VAPB	rabbit	#14477-1-AP, Proteintech	Western blotting<br>immunofluorescence	1:1,000<br>1:200
α-VAPB	mouse	#66191-1-Ig, Proteintech	immunofluorescence<br>PLA	1:200<br>1:100
α-ACBD5	rabbit	#21080-1-AP, Proteintech	Western blotting<br>immunofluorescence	1:1,000<br>1:100
α-OSBPL9	rabbit	#11879-1-AP, Proteintech	Western blotting<br>immunofluorescence	1:1,000<br>1:1
α-TMEM43	rabbit	#ab184164, Abcam	Western blotting	1:1000
α-NUP153	rabbit	# HPA027897, Sigma-Aldrich	immunofluorescence	1:100
α-GAPDH	rabbit	#10494-1-AP, Proteintech	Western blotting	1:5000
α-Nup214	rabbit	Kehlenbach's lab	immunofluorescence	1:100
α-VAPA	rabbit	#15275-1-AP, Proteintech	immunofluorescence	1:200
α-mAB414	mouse	#MMS-120R, Covance	immunofluorescence	1:500
α-SQSTM1	rabbit	#18420-1-AP, Proteintech	Western blotting<br>immunofluorescence<br>PLA	1:1000<br>1:200<br>1:500
α-TIA1	mouse	#sc-101267, Santacruz Biotechnology	Western blotting	1:250
α-LAP2β	rabbit	#06-1002, Millipore	immunofluorescence	1:100
α-ELYS	rabbit	# HPA031658, Sigma	immunofluorescence	1:100
α-Nup358	rabbit	Melchior's lab	immunofluorescence	1:50
α-FUS	rabbit	#HPA008784, Sigma	immunofluorescence	1:100
α-TDP43	rabbit	#10782-2-AP, Proteintech	immunofluorescence	1:500
α-LC3	rabbit	# PM036, MBL International	immunofluorescence	1:100
α-LAMP1	rabbit	#L1418, Sigma	immunofluorescence	1:100
α-HA	mouse	#MMS-101P_HA.11 Clone 16B12, Covance	immunofluorescence	1:500
α-HA	rabbit	# 11867423001, Sigma	immunofluorescence	1.500
α-Na^+/K^+ ATPase	mouse	#sc-21712, Santacruz Biotechnology	Western blotting	1:200
**Secondary antibodies**				
α-rabbit-HRP	donkey	#711-035-152, Jackson ImmunoResearch Laboratories, West Grove, PA, USA	Western blotting	1:5,000
α-rat-HRP	goat	#112-035-003, Jackson ImmunoResearch Laboratories, West Grove, PA, USA	Western blotting	1:10,000
IRDye® 800CW α - rabbit IgG (H + L)	donkey	LI-COR Biosciences	Western blotting	1:10,000
IRDye® 800CW α-mouse IgG (H +	donkey	LI-COR Biosciences	Western blotting	1:10,000

L)				
α-rabbit-Alexa Fluor 488	donkey	#A-21206, Molecular Probes, Eugene	immunofluorescence	1:1,000
α-mouse-Alexa Fluor 488	donkey	#A-21202, Thermo Fisher Scientific	immunofluorescence	1:600
α-mouse-Alexa Fluor 594	donkey	#A-21203, Thermo Fisher Scientific	immunofluorescence	1:750
α-rabbit-Alexa Fluor 594	donkey	#A-21207, Thermo Fisher Scientific	immunofluorescence	1:750
α-rabbit-Alexa647	donkey	#A-31573, Thermo Fisher Scientific	immunofluorescence	1:500
